# Application of Metabolomics in Carcinogenesis and Cancer Prevention by Dietary Phytochemicals

**DOI:** 10.1007/s40495-025-00396-0

**Published:** 2025-02-06

**Authors:** Rebecca Mary Peter, Xiaoyang Su, Ah-Ng Kong

**Affiliations:** 1 Graduate Program in Pharmaceutical Science, Ernest Mario School of Pharmacy, Rutgers, The State University of New Jersey, Piscataway, NJ 08854 USA; 2 Department of Pharmaceutics, Ernest Mario School of Pharmacy, Rutgers, The State University of New Jersey, 160 Frelinghuysen Road, Piscataway, NJ 08854 USA; 3 Metabolomics Shared Resource, Rutgers Cancer Institute of New Jersey, New Brunswick, NJ 08903 USA

**Keywords:** Metabolomics, Metabolomic workflow, Metabolic alterations, Liquid chromatography – mass spectrometry, Phytochemicals, Cancer prevention

## Abstract

**Purpose of Review:**

In this review article, specific emphasis is on evolution of metabolomics in cancer research, metabolomics workflow, general understanding of liquid chromatography – mass spectrometry (LC-MS) based platform for quantitation of metabolites, their biological interpretation and the application in carcinogenesis and cancer prevention by dietary phytochemicals.

**Recent Findings:**

Metabolomics is increasingly becoming a preferred approach for next generation metabolic screening and has profound impact on medical practice. Metabolomics describes the end products of biochemical processes which are greatly influenced by genetic and environmental factors. Metabolic alterations can be linked to potential biochemical reactions/enzymes and their corresponding genes. Thus, these results can be further validated via multi-omics approach including genomics, transcriptomics and proteomics. However, challenges exist within and between omic-domain data integration considering complex biochemical regulation including organism versus tissue versus cellular level processes, epigenetics, transcriptional and post translational modifications. Metabolomics can reflect the steady state or dynamic state of metabolism because metabolites are highly dynamic in space and time.

**Summary:**

Metabolomic analysis of biological samples exhibit the possibility to determine mechanism of action of anti-cancer agents, biomarker discovery and impact of genetic alterations.

## Introduction

**Fig. 1:**
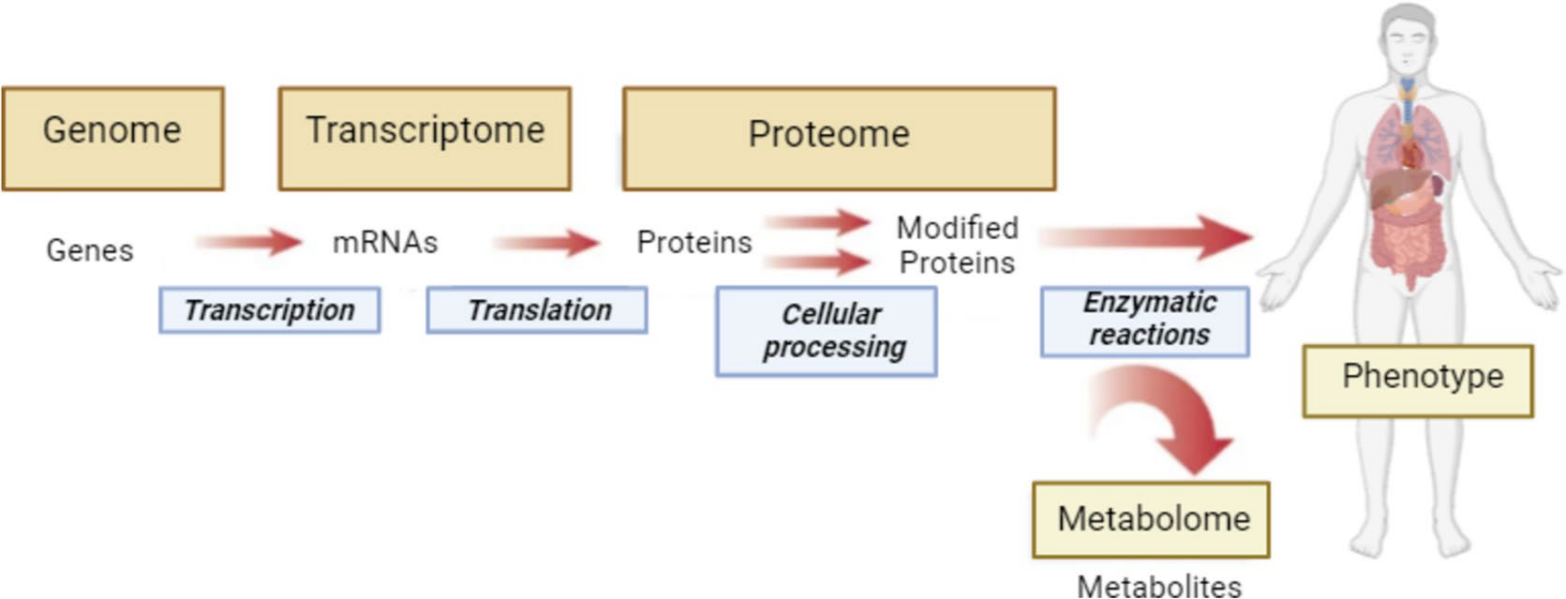
Metabolome represents ultimate endpoint of biological cascade linking genome, transcriptome and proteome. Adapted from (Gerszten & Wang,2008, *Nature, 451(7181), 949–952*)

Metabolomics is an “omic” science which utilizes low molecular weight (50–1500 Da) endogenous metabolites or metabolite profiles present in a biological system to provide insights about the pathophysiology and progress of a disease, and identify potential biomarkers [1]. It allows simultaneous and relative quantification of thousands of different metabolites within a given sample using sensitive and specific methodologies. Since metabolites are final downstream products of genome, transcriptome and proteome, they closely represent the phenotype of an organism at a specific time (Fig. 1). So, studying metabolic differences between unperturbed and perturbed metabolic pathways is likely to create opportunities for biomarker discovery, disease diagnosis and treatment [2]. The potential of this approach is enormous, particularly because minimal biological preparation is necessary. Consequently, it has profound impact on medical practice. It is worth noting that clinicians are able to utilize a patient’s metabolic state to closely represent his/her health status. Very common examples include (i) cardiovascular health assessment based on high density lipoprotein/low density lipoprotein ratio (ii) glucose measurement to monitor diabetes (iii) information on kidney function based on blood urea nitrogen (BUN) and creatinine (iv) diagnosis of potential inborn errors of metabolism in neonates by measuring a panel of metabolites [3]. With growing focus on early medical intervention and disease prevention and the need for personalized medicine, comprehensive study of molecular phenotype associated with lifestyle, nutrition and pathological processes that encompasses substrates, intermediates, and products of biochemical reactions can be accomplished by metabolomic analysis [4–6]. Although, personalized metabolomics has promising perspectives, there are no Food and Drug Administration (FDA)-approved metabolomics tests yet [7].

## Evolution of Metabolomics in Cancer

Metabolomics was first introduced by Stephen George Oliver in his review article on yeast functional genomics published in 1998, and now its potential utility is widely recognized in all of the systems biology [8, 9]. Metabolomics significantly revolutionized the field of cancer research. It is identified as one of the most powerful omics techniques to effectively detect metabolite levels altered by neoplastic progression in a biological sample, opening doors for biomarker identification, drug discovery or development, clinical toxicology, nutritional studies, and quantitative phenotyping [10, 11]. Most cancer cells are found to adapt to hypoxic environment or mitochondrial damage within tumors via metabolic reprogramming of glycolysis leading to lactic acid accumulation. This phenomenon is well known as Warburg effect [12]. In addition, numerous metabolic pathways that affect cell growth and proliferation of cancer cells including energy, lipid or nucleotide metabolism are identified in cancer cells [13]. In 2007, the completion of the first draft of the human metabolome was one of the biggest achievements in the field. The Human metabolome database (HMDB) was designed and linked to other databases including KEGG, PubChem, MetaCyc, UniProt, and GenBank, thus integrating chemical data, clinical information and molecular biology/biochemistry models. The database is known to contain information on more than 220,000 metabolites – both water-soluble and lipid-soluble [14]. A major application of metabolomics research has been cancer biomarker discovery or determining patterns of cancer. Different types of samples besides organ tissues such as serum [15–17], plasma [18], saliva [19], urine [20, 21] and breath [22] have been analyzed for cancer biomarker discovery. In vivo imaging techniques is also an important application of metabolomics for cancer diagnosis [23]. For instance, positron-emission tomography (PET) images tumors based on glucose uptake by tumor cells as most tumor cells are believed to have more glucose uptake than normal cells. PET is done using a radiolabeled glucose analogue, 2-[18F]fluoro-2-deoxy-D-glucose (FDG) [24, 25]. Cancer drug therapies have seen the use of metabolites which became popular soon after second world war. They are called *antimetabolites.* They are chemically similar to endogenous metabolites and have the capability to interfere with normal metabolism of cancer cells, thereby inhibiting cell proliferation. Examples of clinically approved antimetabolites include folate analogs (aminopterin and methotrexate), purine analogs (mercaptopurine) and pyrimidine analogs (fluorouracil, gemcitabine, capecitabine) [26–28].

## Metabolomics Workflow

**Fig. 2:**
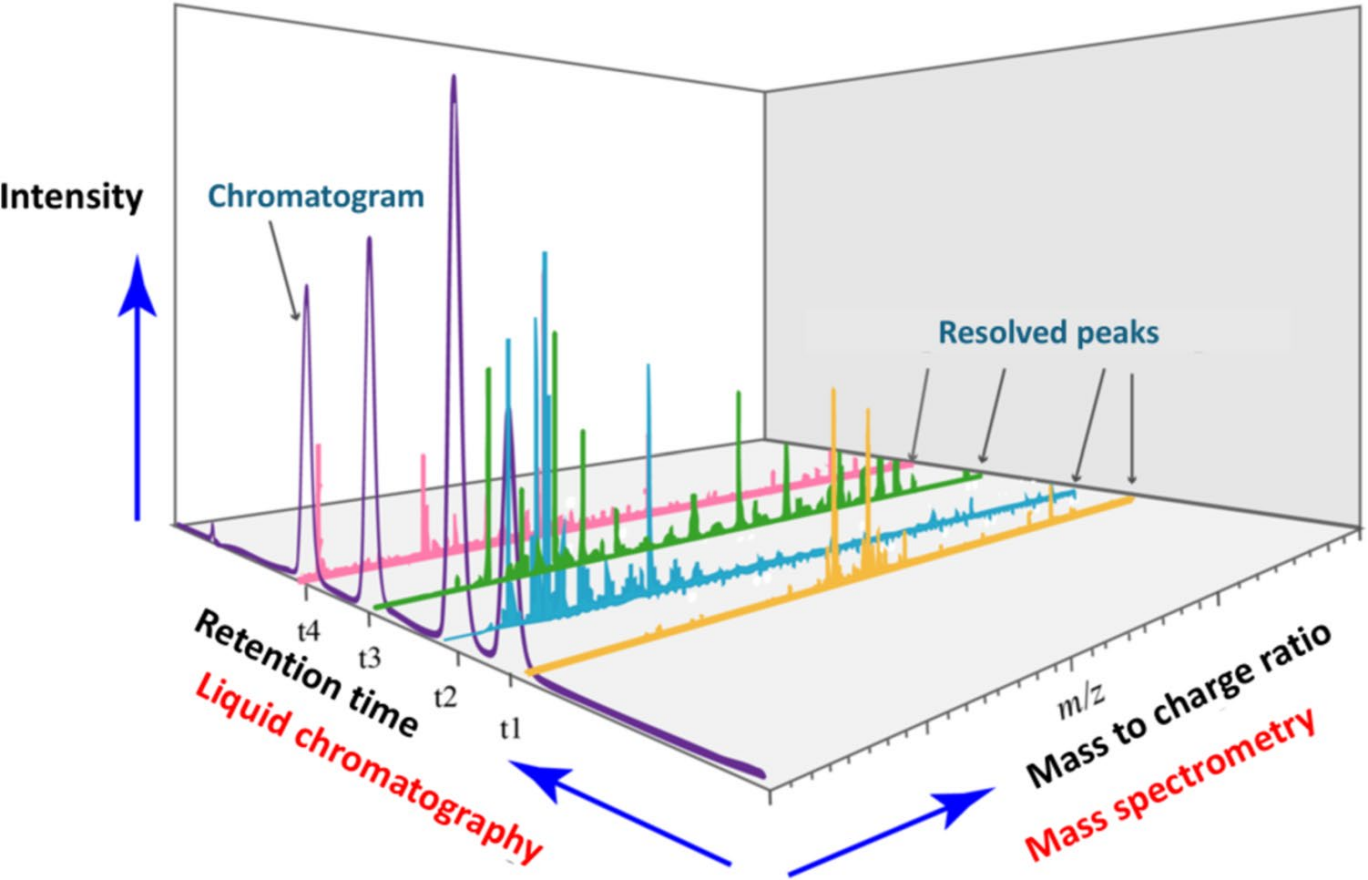
Three dimensional representation of LC–MS data structure

Systematic quantitative characterization of metabolites in a biological sample such as biofluids (serums, urine), cells and tissues, provides terrific platform for translational and clinical research, and diagnostic applications. It involves different techniques to isolate different groups of metabolites from the biological specimen. Sample pre—processing is generally carried out by metabolic quenching, centrifugation and filtration. Samples are then subjected to immediate freezing and sometimes stored as small aliquots to minimize number of freeze–thaw cycles. Analytical workflow consists of sample processing steps including metabolite extraction, chemical derivatization, adjusting solvent/pH, dilution or concentration, spiking reference standards or reference control samples, not necessarily in the same order. Metabolomics data is generally acquired by two main platforms mass spectrometry (MS) and nuclear magnetic resonance (NMR) spectroscopy [29]. Each platform has its own advantages and disadvantages. MS-based metabolomics is preceded by separation techniques such as liquid chromatography (LC) or gas chromatography (GC). It acquires spectral data based on mass-to-charge ratio (m/z) and relative intensity of the ionized compound. NMR acquires metabolomic data by utilizing the principle of energy absorption and re-emission by atomic nuclei based on variations in an external magnetic field. Unlike MS, NMR does not require extensive sample preparation. However, NMR has poor dynamic range and lower sensitivity i.e. lower concentrations of compounds of interest can be masked by larger peaks and cannot be identified. The acquired data is then analyzed to identify and quantitate known/unknown metabolites which are finally studied to determine significant changes between different experimental groups [30–32] (Fig. 2). In this review, specific focus is on pharmaceutical applications of LC–MS based metabolomics.

### Understanding LC–MS Based Metabolomics in Biomedical Research

**Fig. 3:**
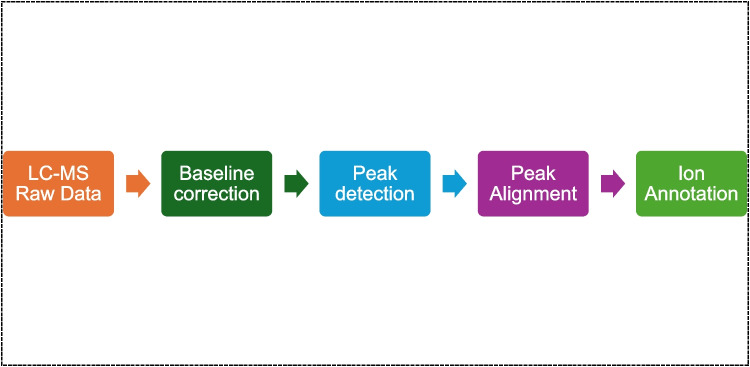
Basic LC–MS based metabolomic data pre-processing workflow

LC–MS based metabolomics is often considered the most popular metabolomics strategy due to its high throughput, soft ionization and good metabolite coverage [33]. This platform scans a biological sample by a mass spectrometer consecutively during chromatography and generates a time series of spectra which represents ions mass to charge ratio (m/z) and intensity values. Processing of this data reports a quantitative value per metabolite feature per sample. This is regarded as a proxy for the sample’s biological metabolite concentration (Fig. 3).

Typically, metabolic analysis is categorized as (i) targeted and (ii) untargeted. The targeted approach is usually hypothesis driven wherein known metabolites such as substrates of an enzyme, downstream products of a protein, a particular metabolite class or a component of a metabolic pathway are identified and quantitated. On the other hand, untargeted approach helps to generate new hypothesis for further experiments by determining all the metabolites of a biological system. This method screens potential and putative metabolites of interest which are then subjected to functional interpretation and pathway analysis [33]. For example, elucidating metabolic alterations in biological samples based on anti-cancer drug treatment or genetic alteration. Following sections will briefly discuss basic bioinformatic steps involved in analyzing metabolites extracted from biological specimens.

#### Data Acquisition

This is the first step to discover metabolic profile, once the metabolites are extracted. The mass spectrometer consists of three major parts: ion source, mass analyzer and detector. The ion source converts sample molecules into ions via techniques like electrospray ionization (ESI) and these ions are resolved by mass analyzer before they are measured by detector. Considering diverse chemical properties of metabolites, biological sample needs to be analyzed in both positive and negative ionization modes under scan range of m/z 50–1000 to ensure maximum metabolome coverage. Typically, three data types are acquired. They are (a) chromatographic retention time of intact metabolite (b) MS1: m/z of intact molecule (c) MS/MS or MS^n^ m/z of fragmented metabolite [33].

#### Data Pre-Processing

Once the raw LC–MS data is generated, it is important to convert it into a format that can be easily interpreted and reproduced. The LC–MS data pre-processing consists of chromatographic peak detection, sample alignment and peak correspondence. First and foremost, LC–MS data are *screened for possible outliers* or peaks that show undesired deviation from the majority of their replicates (analytical or biological). Very often, principal component analysis (PCA) are used to identify sample outliers. *Filtering methodologies* are adopted to suppress the noise/contaminants while preserving the peaks in the data. *Baseline correction* algorithms are used to prevent over-estimate of the ion intensities with increasing retention times. This is normally done by estimating the low frequency baseline and then subtracting the estimated baseline from the raw signal. After that, *peak detection* is performed on each extracted ion chromatogram (EIC) to detect the peak(s). EIC is a 2-dimensional signal of intensity versus retention time over a small m/z interval. The next step is *peak matching and retention time alignment* which is done to correct for the retention time drift and make sure that the same ion is compared across samples. *Ion annotation* is used to identify the peaks originating from the same metabolite. Finally, relative abundance of endogenous metabolite ion intensities is *normalized* by internal standards. Pre-processed LC–MS data then becomes suitable for subsequent statistical analysis [33]. Numerous software tools are available to support LC–MS data pre-processing, including those provided by most instrument manufacturers as well as publicly available ones. For example Progenesis QI (Waters), AnalyzerPro XD (Spectral Works), Sieve (Thermo), Compound discoverer (Thermo), MetaboScape (Brucker), XCMS 15,16,17, MZmine 18, MetAlign 19 and MAVEN 20 [34].

#### Statistical Analysis

Main objective of statistical analysis is to determine significantly altered peak intensities between distinct biological groups. To assess the statistical significance of each peak, univariate or multivariate analysis is done depending on the experimental design. P-values are usually considered in univariate approaches. But in multivariate untargeted study scenarios, there is high possibility of false discovery even with a small p-value threshold due to simultaneous measurement of thousands of metabolites. A q-value for each peak is estimated to determine the chance of false discovery at a given test statistics threshold. Furthermore, several supervised learning techniques are used to build models that can predict the annotations for new samples [33, 35]. MetaboAnalyst is a very popular integrated software for statistical analysis of LC–MS data [36].

#### Metabolite Identification, Verification and Quantitation

In untargeted metabolic analysis, endogenous metabolite identification is considered one of the key challenges in metabolomic studies. It is mainly achieved via mass-based search and manual verification. Metabolites are combination of elements like ‘C’, ‘H’, ‘O’, ‘S’, ‘N’ or ‘P’. Their chemical and physical diversities make their identification based on MS data difficult. Firstly, m/z value of metabolite ion of interest having molecular mass within the pre-specified tolerance of the query masses is retrieved from appropriate database(s) [37–39]. However, due to the existence of isomers and the limited accuracy of mass spectrometers, putative identifications from mass-based search are not always unique [40]. Improved approaches like isotope labelling methods can minimize ambiguities from the mass-based search. But unique identification cannot be guaranteed [41]. Mass-based search results of putative identifications can be verified by comparing the retention times or tandem MS spectra of authentic compounds with ions of interest. For more confidence on identification of some metabolites, MS can be extended from MS^2^ to MS^3^ or MS^4^ to acquire further fragmentation information [33]. To understand metabolic alterations in response to disease, treatment and genetic changes, quantitation of metabolites in biological samples is an important aspect of metabolomics. The two main metabolite quantitation methods are triple quadrupole (QqQ)-based selected reaction monitoring (SRM) and ion trap; and high resolution mass spectrometry (HRMS)-based full-scan MS analysis. The former is applicable to targeted metabolites wherein the precursor ion in the first quadrupole (MS1) is fragmented into daughter ions, of which specific daughter ion is selected for fragmentation in the second quadrupole (MS2). Absolute quantitation of metabolite concentration is determined by correlating signal intensities of analytes of interest with a calibration curve set up using spiked stable isotope-labeled analogues [42]. HRMS is an untargeted metabolomic approach which can virtually determine all compounds present in a sample. It operates in full scan mode. To optimize detection sensitivity, metabolites coverage and quantitation accuracy by HRMS, more focus is on sample preparation and LC separation [33].

#### Functional Interpretation and Pathway Analysis

To get meaningful picture about the investigated biological system, metabolite set enrichment analysis (MSEA) has been developed to deduce functional interpretation of a selected set of metabolites, after verification and quantitation of metabolites from untargeted studies [43]. MSEA utilizes publicly available bioinformatic resources (like HMDB and KEGG) and text mining of literatures to perform enrichment analysis on a list of altered metabolites from a biological specimen. This along with pathway enrichment analysis forms a powerful basis for identification of a biological pathway or disease condition, which can be further investigated [33, 44].

## Application of Metabolomics in Carcinogenesis and Cancer Chemopreventive Phytochemicals

For more than a decade, we determined epigenetic changes and potential therapeutic targets associated with phytochemicals with focus on Keap1-Nrf2 signaling pathway in several cancer cell lines and in vivo models, particularly in the early stages of carcinogenesis [45–51]. Understanding the relationship between cellular metabolism and chromatin remodeling or epigenetic reprogramming [52, 53], we explored phytochemical intervention in cellular metabolism and identified biological effects on metabolic co-factors in the context of oxidative stress [54]. For example, ursolic acid, a triterpenoid phytochemical exhibited anticancer chemopreventive effect in prostate cancer xenograft and PTEN (phosphatase and tensin homologue deleted on chromosome 10) knockout mouse by regulating metabolic rewiring while driving epigenetic CpG methylation reprogramming, and transcriptomic signaling [55, 56]. We also elucidated benzo[a]pyrene (B[a]P) driven epigenetic and metabolic alterations in the initiation, promotion and progression of non-melanoma skin cancer (NMSC) in SKH-1 hairless mice which was found to be intercepted by ursolic acid by modulating cancer associated metabolisms of thiamin, ascorbate, pyruvate and citrate as well as biosynthesis of beta-alanine and pathothenate coenzyme A (CoA) [57, 58]. Organosulphur compound, diallyl sulphide was shown to exhibit chemopreventive effects via metabolic alterations in early stages of sequential lung carcinogenesis induced by cigarette smoking carcinogen 4-[methyl(nitroso)amino]−1-[3-pyridinyl]−1-butanone (NNK) in A/J mouse model [59]. Fucoxanthin, a carotenoid with many antioxidant/anti-inflammatory properties suppressed tumor promoter, TPA-induced oxidative stress mediated skin cell transformation by alteration of cellular metabolites [60]. Butyrate, a short-chain fatty acid produced by dietary fiber and a well known inhibitor of histone deacetylases (HDACs) activated mitochondrial tricarboxylic acid (TCA) cycle, but inhibited methionine metabolism both of which are tightly coupled to the epigenetic machinery, eliciting cancer-preventive effects in colorectal cancer (CRC) HCT116 cells [61]. Sulforaphane, a potent Nrf2 activator attenuated UVB-induced skin damage associated aberrations and showed potential linkage among the metabolome, CpG methylome, and transcriptome of human skin keratinocytes (HaCaT cells) [62]. Furthermore, we extended our interest in understanding metabolic implications of HDAC inhibition based on treatment of FDA approved drugs – vorinostat and belinostat in lung cancer, including alteration in mitochondrial metabolism in our recent publications [63, 64].

## Summary

Metabolomics has opened novel avenues for biomedical research. Metabolic biomarkers can be widely utilized and studied in clinical setting. Based on this review, there is an effort to discuss the possibility of studying metabolic reprogramming in in-vitro and in-vivo disease as well as transgenic models.

## Data Availability

No data were generated and incorporated in the current study.

## Abbreviations

B[a]P: Benzo[a]pyrene
BUN: Blood urea nitrogen
CoA: Coenzyme A
CRC: Colorectal cancer
EIC: Extracted ion chromatogram
ESI: Electrospray ionization
FDA: Food and Drug Administration
FDG: 2-[18F]fluoro-2-deoxy-D-glucose
GC: Gas chromatography
HDACs: Histone deacetylases
HMDB: Human metabolome database
HRMS: High resolution mass spectrometry
LC: Liquid chromatography
MS: Mass spectrometry
MSEA: Metabolite set enrichment analysis
NMR: Nuclear magnetic resonance
NMSC: Non-melanoma skin cancer
NNK: 4-[Methyl(nitroso)amino]-1-(3-pyridinyl)-1-butanone
PCA: Principal component analysis
PET: Positron-emission tomography
PTEN: Phosphatase and tensin homologue deleted on chromosome 10
QqQ: Triple quadrupole
SRM: Selected reaction monitoring
TCA: Tricarboxylic acid
